# Pathophysiology and Therapeutic Approaches for Spinal Cord Injury

**DOI:** 10.3390/ijms232213833

**Published:** 2022-11-10

**Authors:** Rui Lima, Andreia Monteiro, António J. Salgado, Susana Monteiro, Nuno A. Silva

**Affiliations:** 1 Life and Health Sciences Research Institute (ICVS), School of Medicine, University of Minho, 4710-057 Braga, Portugal; 2 ICVS/3B’s Associate Laboratory, PT Government Associated Laboratory, 4806-909 Braga/Guimarães, Portugal

**Keywords:** spinal cord injury, molecular therapies, neuroprotection, neuroregeneration

## Abstract

Spinal cord injury (SCI) is a disabling condition that disrupts motor, sensory, and autonomic functions. Despite extensive research in the last decades, SCI continues to be a global health priority affecting thousands of individuals every year. The lack of effective therapeutic strategies for patients with SCI reflects its complex pathophysiology that leads to the point of no return in its function repair and regeneration capacity. Recently, however, several studies started to uncover the intricate network of mechanisms involved in SCI leading to the development of new therapeutic approaches. In this work, we present a detailed description of the physiology and anatomy of the spinal cord and the pathophysiology of SCI. Additionally, we provide an overview of different molecular strategies that demonstrate promising potential in the modulation of the secondary injury events that promote neuroprotection or neuroregeneration. We also briefly discuss other emerging therapies, including cell-based therapies, biomaterials, and epidural electric stimulation. A successful therapy might target different pathologic events to control the progression of secondary damage of SCI and promote regeneration leading to functional recovery.

## 1. Spinal Cord Anatomy and Physiology

The spinal cord is the major communication channel between the body and the brain. Additionally, the spinal cord can independently respond to sensory information without input from the brain through reflex arcs and produce repetitive patterns of motor behavior using self-containing circuits known as Central Pattern Generators (CPGs). The spinal cord extends from the base of the brain in the medulla oblongata through the foremen magnum of the skull to the L1/L2 lumbar vertebra, where it terminates as the conus medullaris. Distal to this end of the spinal cord is a collection of nerve roots called the cauda equina. Like the brain, which is protected by the cranium, the spinal cord is likewise protected by a bone structure called vertebral column. The spinal cord is also protected by three membranes of connective tissue called meninges (dura mater, arachnoid mater, and pia mater). The subarachnoid space (between arachnoid and pia), which is filled with cerebrospinal fluid, and the epidural space (between dura and periosteum), which is filled with loose fibrous and adipose connective tissues, also helps to protect the spinal cord [1,2,3].

Contrary to the brain, the spinal cord’s gray matter is centrally surrounded by white matter. The gray matter comprises neuronal cell bodies, interneurons, dendrites of efferent neurons, entering fibers of afferent neurons, neuroglia cells, and axons which are predominantly unmyelinated. Unlike gray matter, white matter is a collection of interconnecting fibers composed mostly of myelinated sensory and motor axons. The surrounding white matter is composed mostly of groups of myelinated axons [1,3,4].

The spinal cord has numerous groups of nerve fibers going towards and coming from the brain. The tracts are described according to the funiculus within which they are located. The ascending tracts usually start with the prefix spino- and end with the name of the brain region where spinal cord fibers first synapse (e.g., spinothalamic tract). The descending motor tracts begin with the prefix denoting the brain region that gives rise to the fibers and ends with the suffix -spinal (e.g., corticospinal tract) [1,3,5].

The spinal nerves leave and enter the spinal cord by the ventrolateral and dorsolateral sulcus. Sensory neurons enter the spinal cord by the dorsal side, while axons of efferent (motor) neurons leave the spinal cord on the ventral side via the ventral roots. Near the cord, the dorsal and ventral roots from the same level combine to form a spinal nerve on each side of the spinal cord. The spinal nerves’ nomenclature depends on the vertebral levels from which they exit: cervical (C1–C8), thoracic (T1–T12), lumbar (L1–L5), sacral (S1–S5), and coccygeal (Co1). Cervical nerves are responsible for controlling the muscles and glands, and receive sensory input from the neck, shoulder, arm and hand. Thoracic nerves are associated with the chest and abdominal walls, and the lumbar nerves carry information related to the hip and leg. The sacral nerves are associated with the genitals and lower digestive tract control, and, finally, the coccygeal nerve supplies the skin over the coccyx [1,3].

## 2. Spinal Cord Injury

A spinal cord injury is a devastating event that leads to motor, sensory, and autonomic dysfunctions. The complexity of this event and the lack of an effective treatment make SCI a worldwide problem. A study performed by the Global Burden of Diseases, Injuries and Risk Factors (GBD) reported 0.93 million (0.78–1.16 million) new SCI cases globally, with a prevalence of 27.04 million cases (24.98–30.15 million) [6]. The annual incidence of SCI varies greatly from region to region: 7 to 37 cases per 100,000 individuals [6]. In the United States, traffic accidents are currently the leading cause of injury (38%), followed by falls (30%), violence (13.5%), and sports/recreation accidents (9%) [7]. The average age at injury is 42 years, and 80% of spinal cord injuries occur in males [7].

The American Spinal Cord Injury Association (ASIA) Impairment Scale defines the degree of neurological loss as: A (complete sensorimotor loss below the lesion including absent sacral sensation), B (sensory but no motor function below the lesion level), C (some motor preservation, but the majority of muscles are less than 3), and D (muscle grade is 3 or greater in the majority of groups below the lesion) [8]. Incomplete tetraplegia is currently the most frequent injury (45%), followed by incomplete paraplegia (21.3%), complete paraplegia (20%), and complete tetraplegia (13.3%) [7]. Less than 1% of persons experience complete recovery by hospital discharge [7].

### 2.1. Primary Injury

SCI results from an insult that damages the spinal cord, which can be subdivided into non-traumatic and traumatic. Non-traumatic injury occurs when an acute or chronic disease, such as a tumor, infection, or degenerative disease, causes damage to the spinal cord. The traumatic and most common SCI results from a traumatic impact that fractures or dislocates vertebrae. The initial mechanical impact to the spinal cord at the time of injury is denominated primary injury. The most common form of primary impact is compression injury, which typically occurs through burst fractures, with bone fragments compressing the spinal cord, or through fracture-dislocation injuries [9,10]. Impact alone with transient compression is observed less frequently, but most commonly in hyperextension injuries [9]. Distraction injuries occur when two adjacent vertebrae are pulled apart, causing the spinal column to stretch and tear in the axial plane [9]. Lastly, laceration and transection injuries can occur through projectile injuries, severe dislocations, or sharp bone fragment dislocations, and can present high variability from minor injuries to complete transection [9]. Regardless of the form of primary injury, these forces will directly damage the neurons, glial cells, and the neurovasculature of the spinal cord [9]. Overall, the extent of the primary injury and its level determines the severity and outcome of SCI [11].

### 2.2. Secondary Injury

Following the primary injury, a derived degenerative process initiates within minutes and hours, which is proportional to the magnitude of the initial insult. This resultant process is commonly denominated by secondary injury. This comprises permeability and vascular alterations, ionic disruption and glutamate excitotoxicity, metabolic alterations, a dysfunctional inflammatory response, and initiation of glial scarring [12]. The main tissue alterations promoted by the secondary injury cascade of events are described below.

#### 2.2.1. Permeability and Vascular Alterations

The hemorrhage associated with the “primary injury,” coupled with systemic hypotension, culminates in a major reduction in the blood flow at the lesion site [13]. Over time, the decreased blood flow leads to ischemia. Although it remains unclear, the retraction of the blood supply may be due to microvascular detriment, hypotension, loss of autoregulation, and an increase in interstitial pressure [14]. Ultimately, cells are deprived of oxygen and glucose, leading to necrosis [15]. Moreover, post-injury hemorrhage and ischemia also impact blood–spinal cord barrier (BSCB) permeability. The direct alteration of BSCB endothelial cells promotes the infiltration of immune mediators that leads to edema, and might increase the pro-inflammatory environment in the injured spinal cord [16].

#### 2.2.2. Ionic Disruption and Glutamate Excitotoxicity

After the insult, the homeostatic ionic balance is severely compromised. Membrane depolarization and ATPase disruption enhance neuronal and glial cell death by increasing intracellular calcium (Ca^2+^) levels. Additionally, there is an exacerbated release of glutamate to the extracellular space, reaching neurotoxic levels [17]. Glutamate is a well-described excitatory neurotransmitter, regulated by Ca^2+^ flux at the synaptic cleft. After SCI, there is an excessive release of this amino acid [14,17], and consequently, excessive activation of glutamate receptors (NMDA and AMPA) that leads to an increase in sodium (Na^+^) and Ca^2+^ influx. Ionic dysregulation directly impacts neuronal and glial cells, especially oligodendrocytes and neurons, leaving them vulnerable to cell death. In addition, axonal degeneration is mediated by Ca^2+^ influx from the endoplasmic reticulum (ER) through the inositol triphosphate (IP3) receptor, which promotes mitochondrial permeability [18]. Overall, glutamate excitotoxicity disturbs ionic homeostasis and normal mitochondrial functioning, resulting in axonal demyelination and neuronal loss at the injury site [12,19].

#### 2.2.3. Metabolic Alterations

Ischemia, oxygen deprivation, and oxidative stress lead to the production of high levels of reactive oxygen species (ROS) and reactive nitrogen species (RNS) [14,20]. As a consequence, ROS and RNS are strongly reactive with polyunsaturated fatty acid of the cellular membrane, leading not only to lipid peroxidation, but also to damage at the protein and nucleic acid levels. Furthermore, the formation of free radicals also invokes architectonic alterations of the cytoskeleton and organelle membranes, mitochondrial dysfunction, and increased intracellular Ca^2+^ uptake [9,14].

The formation of specific aldehyde products promotes cell membrane disruption, affecting nearby healthy cells. Additionally, impairments at the metabolic levels are also observed in the normal functioning of the transmembrane (Na^+^/K+)-ATPase enzyme. As a major Ca^2+^ pump, ATPase is crucial for maintaining neuronal excitability and alterations in its activity, triggering axonal and neuronal loss [9,19].

#### 2.2.4. Inflammatory Response

Inflammation is a major “secondary injury” event, and its dysregulated nature leads to more neuronal damage [21]. Initiation of the “secondary injury” leads to cell activation of astrocytes, fibroblasts, pericytes, and microglia. The BSCB disruption further drives injury progression by facilitating the infiltration of non-resident cells. Peripheral immune cells invade the injury site and chronically persist within the spinal cord [22]. Fibroblasts, which infiltrate from the periphery or differentiate from other resident cells, deposit inhibitory extracellular matrix (ECM) components that aggravate the inflammatory environment [23]. Moreover, SCI generates cellular debris and releases intracellular proteins that induce potent inflammatory stimuli. This debris signal, also called damage-associated molecular patterns (DAMPs), is usually hidden from immune surveillance within the intact CNS [24,25]. After an injury, DAMPs engage pattern recognition receptors (PRR) of inflammatory cells involved in foreign microbe detection [26]. As a result of the rapid DAMP- and PRR-mediated activation, resident and peripheral inflammatory cells are recruited to the lesion site [24,25]. Consequently, these cells release various oxidative stress regulators, cytokines, chemokines, and other inflammatory mediators that exacerbate the inflammatory response [24,27].

Regarding microglia, the cellular morphology and protein expression profiles are altered following SCI. Microglia cells retract their processes and assume an amoeboid morphology, making them better prepared for phagocytosis and debris clearance. Reactive microglia closely resemble circulating macrophages in terms of morphology, protein expression profile, and function [28]. Together with morphological changes, the release of chemokines and cytokines recruits neutrophils and macrophages into the injured spinal cord [29]. The first type of infiltrating immune cells are the neutrophils, which, in rodents and humans, have their peak within the spinal cord around 1-day post-injury [27,30,31]. The by-products produced after neutrophil-mediated phagocytosis create a cytotoxic environment with the production of ROS and reactive nitrogen species (RNS) [32].

Moreover, neutrophils persist chronically at low levels in the spinal cord, but decrease within a week of injury in both rodents and humans [22,33]. Monocyte-derived macrophages also infiltrate the spinal cord [34] and contribute, along with proteolytic enzymes, ROS, and inflammatory cytokines, to the injury microenvironment. They also perform critical functions, such as debris clearance, cellular remodeling, and production of pro-regenerative factors [28,35]. Likewise, CNS reactive B- and T-cells also infiltrate the spinal cord, and have been suggested to promote axonal injury and demyelination [9]. Recently, single-cell transcriptomic analyses revealed that even in the chronic phase, the major cell types of the spinal cord are still considerably deviated from uninjured states, and that microglia were the most dynamically altering cell population after SCI. Even by day 42, when tissue homeostasis is stabilized, the microglial populations diverge from those before the injury, indicating the long-lasting alterations in the immune microenvironment after injury [36]. It is important to point out, as the more we understand the practical role of the inflammatory response, the more obvious it becomes that this response can support both beneficial and detrimental effects on recovery. Indeed, an acute inflammatory response is needed and crucial for successfully repairing the injured tissue [37]; however, after this initial response, it is important to resolve the inflammatory response by the complex orchestration of different cells and molecular events [38].

#### 2.2.5. Inhibitory Environment

The regeneration of CNS following injury is reduced due to multiple inhibitory factors at the injury site. Several researchers have shown that there is an initial growth response following injury; however, once axons encounter this inhibitory environment, the growth is blocked, leaving dystrophic axonal end bulbs in their place [39]. Within the CNS, cells are surrounded by an ECM composed of a complex and interactive network of glycoproteins, proteoglycans, and glycosaminoglycans [40]. Under different circumstances, these molecules can either promote neurite outgrowth, such as during neuronal development [41], or inhibit it, such as after injury [42] or after neural degeneration [43].

Axonal retraction occurs in two phases: an early axon intrinsic, cytoskeleton-associated phase, in which Ca^2+^-dependent activation of calpain proteases leads to cytoskeletal breakdown [44], and a macrophage-dependent phase, in which infiltration of phagocytic macrophages correlates with retraction of dystrophic axons [45]. Alongside this, there is an increase in the number of inhibitory proteins, including myelin-associated inhibitors (MAIs), chondroitin sulfate proteoglycans (CSPGs), as well as growth-inhibiting molecules such as proneurotrophins [46].

Nogo-A, oligodendrocytes myelin glycoprotein (OMgp), and myelin-associated glycoprotein (MAG) have all been identified as MAIs that can collapse axonal growth cones and inhibit neurite outgrowth [47]. Nogo-A was identified as a neurite growth inhibitor in the 1980s [48]. The evidence of inhibitory effects of Nogo-A came from in vitro studies in which exposure of chicken retinal ganglion and rat dorsal root ganglion (DRG) neurons to Nogo-A was shown to inhibit neurite outgrowth and induce growth cone collapse [49,50]. The OMgp is also expressed in oligodendrocytes and several types of CNS neurons, such as pyramidal cells in the hippocampus and Purkinje cells in the cerebellum, among others [51]. Although less is known about OMgp in comparison to Nogo-A and MAG, it has also been shown to be a potent inhibitor of neurite outgrowth in multiple cell lines and primary neuronal cultures [52,53].

MAG is a minor component of mature, compact myelin, enriched in the periaxonal membrane of the myelin sheath, and is expressed by oligodendrocytes and Schwann cells [54]. The inhibitory effect of MAG was found in studies investigating its interaction with primary neurons. Purified recombinant MAG was found to block neurite outgrowth and induce growth cone retraction [55,56]. The inhibitory properties of MAG were further confirmed by Tang and colleagues, demonstrating that myelin from MAG knockout mice was not inhibitory to the growth of DRG neurons in vitro compared to myelin from wild-type mice [57]. Furthermore, inhibition of neurite outgrowth was completely abolished by immunodepletion of MAG from the soluble fraction of myelin-conditioned media [58]. These observations suggest that soluble MAIs, likely released after injury, can influence the growth capacity of neurons and axons in addition to myelin debris.

The ECM of the CNS is rich in CSPGs, some existing within the extracellular milieu and others associated with specific structures. Within the CNS, CSPGs can associate with specialized structures, denominated perineuronal nets (PNNs), which surround the soma and dendrites of mature neurons. The PNNs are ECM proteins including hyaluronan, CSPGs, and linking proteins [59]. There are also a number of CSPGs, such as brevican, neurocan, aggrecan, and versican, which bind to the hyaluronan backbone of the PNN [59]. Maintenance of this specialized structure is important for synaptic and network stabilization and homeostasis. Specifically, PNNs stabilize mature neurons by reducing dendritic spine plasticity [60], forming a scaffold for synaptic inhibitory molecules [61] and also restricting the movement of receptors at the synapse [62]. The formation and maturation of PNNs are concurrent with the development and maturation of the nervous system. After injury, CSPGs are actively secreted into the ECM, mainly by reactive astrocytes [63], but with a minor component secretion by macrophages and oligodendrocytes [64,65,66]. This results in an abundance of CSPGs at the injury site, adding to the inhibitory milieu. The inhibitory effect of the CSPGs is mediated through the protein tyrosine phosphatase sigma (PTPσ) receptor. When CSPGs bind to PTPσ receptors, the GTPase Rho/ROCK signaling pathway is activated. In neurons, this inhibits axonal growth, leading the growth cone into a dystrophic state [42,67,68].

#### 2.2.6. Spinal Cord Scarring

As referenced above, SCI activates astrocytes, pericytes, and fibroblasts, promoting the development of a glial/fibrotic scar. Astrocytes activation and subsequent glial scar boundaries are enhanced by the increase in transforming growth factor-beta (TGF-β) [69,70,71]. TGF-β increases microglia/macrophage and astrocyte activation, as well as fibronectin and laminin deposition [70]. Moreover, the signal transducer and activator of the transcription 3 (STAT3) transcription factor is important in establishing glial scar borders that isolate infiltrating cells into the lesion epicenter [72,73].

The deposition of connective tissue and reactive gliosis creates a physical barrier, providing nonspecific topographical cues which affect cellular migration [74,75]. The removal of some inhibitory ECM components, such as CSPGs, improves neurite growth in vivo. However, the removal of other components, such as collagen, fails to promote regeneration and recovery [76]. Together with the chemical components of the scar, stiffness of the newly created ECM also acts as a physical barrier that inhibits axon growth [76,77]. It is important to note that the role of the scar is complex. Some studies have shown the beneficial effects of the glial scar, namely repairing the BSCB, which restrains the inflammatory response, and toxic species sequestration to the injury site [78,79]. Moreover, astrocytes’ capacity to support axon growth and, therefore, neural plasticity in mammalians has been increasingly documented [80]. Additionally, non-mammalian injury models have highlighted glial bridges’ importance for neuronal regeneration [81,82]. In 2017, Hara and coworkers published a study characterizing astrocytes’ varying phenotypes, specifically regarding a lesion site [83]. In this study, three distinct subtypes of astrocytes associated with the glial scar were characterized: naïve, reactive, and scar-forming astrocytes [83]. Interestingly, when the reactive astrocytes were transplanted into the naïve spinal cord, they reverted to naïve astrocytes; likewise, they converted to scar-forming astrocytes when transplanted into an injury site, demonstrating that the environment dictates astrocytic phenotype and consequently glial scar-mediated inhibition [83]. Given the diversity of astrocytes, the next challenge will be to determine context-dependent astrocyte functions, including their regulation of neuronal repair.

### 2.3. Chronic Phase

Following the secondary injury, the chronic phase is established, and this can lead to the continuous expansion of the lesion site of the patients with SCI. The chronic phase is characterized by scar maturation, cystic cavitation, and axonal dieback [19,84,85]. The process of Wallerian degeneration of injured axons is ongoing, and it may take years for severed axons and their cell bodies to be entirely removed [86]. The lesion may not remain static and syrinx formation may occur, commonly causing dissociated sensory reduction, deterioration of motor function, and neuropathic pain [87,88,89,90].

**Figure 1:**
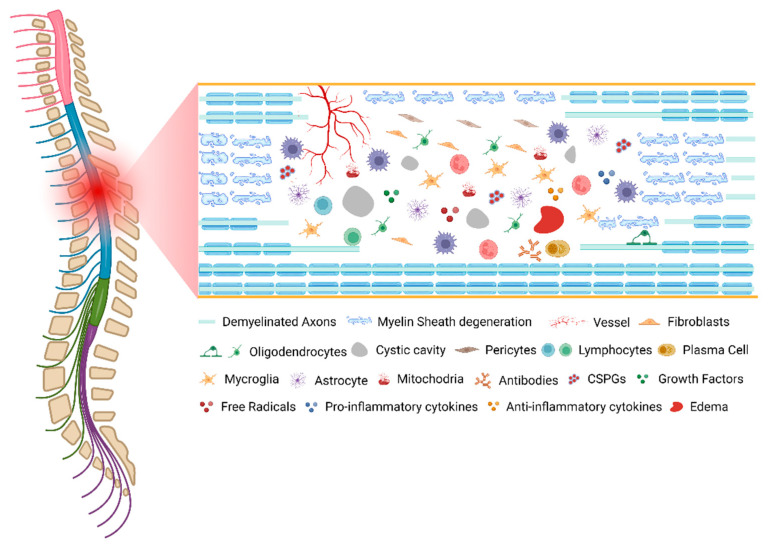
The pathophysiology of spinal cord injury. Created with BioRender.com (accessed on 11 October 2022).

As described, SCI pathophysiology involves many mechanistically distinct processes that interact in order to both limit and enhance recovery following injury (Figure 1). This dichotomy is well demonstrated by the astrocytic response, which serves to reestablish the blood–brain barrier (BBB), restore ionic homeostasis, and limit immune cell infiltration while severely limiting the ability of axons to regenerate and diminishing functional recovery through the formation of the astrocytic scar. An important consideration for understanding the pathophysiology of human SCI is that each injury is unique, both in cause and resultant damage.

## 3. Clinical Management

Currently, the first approach after trauma is the surgical decompression of the spinal cord. Based on a multicenter study—Surgical Timing in Acute Spinal Cord Injury Study (STASCIS)—it was demonstrated that early surgery (<24 h) resulted in better neurological recovery compared to late surgery (≥24 h) in cervical SCI patients [91]. Therefore, the encouraging preclinical and clinical outcomes observed after early decompression show the importance of realigning and relieving the spinal cord’s compressive ligaments. Even with some concerns, surgical intervention shows positive results in the reduction in tissue damage and improvements in neurological outcomes [92,93]. Therefore, the recent AOSpine guidelines and the American Association of Neurological Surgeons (AANS) recommend an early surgical decompression, within the first 24 h [94].

Another immediate priority is stabilizing and controlling the cardiovascular and hemodynamic parameters [95]. During the acute phase of SCI, the pathophysiology is aggravated due to some cardiovascular instability experienced by patients (hypotension, hypoxemia and pulmonary dysfunction). Therefore, the AANS guidelines also recommend tight and continuous hemodynamic monitoring with some preventive interventions, such as the administration of vasopressors [96] and the maintenance of the mean arterial blood pressure (MAP), ranging from 85 and 90 mmHg [97]. In fact, it was shown that prophylactic treatments could be beneficial in lowering the risk of venous thromboembolic events without significantly increasing the risk of bleeding and mortality in acute SCI [98].

Until recently, methylprednisolone sodium succinate (MPSS) was a first-line drug treatment for SCI patients [99]. MPSS is a synthetic glucocorticoid that can interfere with pro-inflammatory cytokine signaling and arachidonic acid metabolites while upregulating the expression of anti-inflammatory factors [100]. A high dose of MPSS has been recommended for the acute management of SCI patients based on the results of three large clinical trials, the National Acute Spinal Cord Injury Studies (NASCIS) [101,102,103,104]. However, several studies reported that MPSS increased the risk of complications such as pneumonia, gastrointestinal hemorrhage, urinary tract infection, wound infection, hyperglycemia, myopathy, and sepsis [100,105]. For these reasons, the American Association of Neurological Surgeons/Congress of Neurological Surgeons and the Food and Drug Administration (FDA) no longer recommend its use for the acute treatment of SCI [106].

Other pharmacological agents, such as naloxone, Monosialotetrahexosylganglioside (GM1), and thyrotropin-releasing hormone (TRH) have been tested in large multicenter clinical trials [107]; however, none have demonstrated strong beneficial effects for SCI patients. The lack of compelling clinical therapies to treat this condition reinforces the importance of pursuing novel therapeutic approaches in order to improve SCI patients’ quality of life.

## 4. Innovative Therapeutic Approaches

Several innovative approaches to achieving relevant functional recovery after SCI have emerged. This review will focus on recent advances in molecular therapies for SCI repair. However, we will also briefly mention some promising approaches based on biomaterials, electrical stimulation, and cellular therapy.

### 4.1. Molecular Therapy

Molecular approaches focus on the modulation of a specific secondary event to promote neuroprotection or neuroregeneration. The analysis of each drug considered the pathophysiological events that the molecule aimed to modulate. A summary of the molecular therapies and their targets can be found in Table 1.

Upon primary injury, vascular disruption occurs, leading to infiltration of blood cells in neuronal tissue and resulting in higher tissue damage. The reduction in vascular alterations in the acute phase or the revascularization of tissue in the chronic phase of injury can be valuable tools to protect or regenerate neural tissue. Molecules such as diazoxide and glibenclamide have been explored in order to modulate this pathological event.

**Diazoxide** is a potassium channel activator commonly used to treat patients with hyperinsulinemic hypoglycemia [108]. In recent years, research has suggested this drug as a potential auxiliary therapy candidate for patients with spinal cord injury. Yamanaka et al. described that Diazoxide administration was responsible for the significant upregulation of STAT3, resulting in preserved neuronal viability, motor function, and protection against intrinsic apoptosis pathway, attenuating spinal cord ischemia-reperfusion injury [109]. Studies in animal models have demonstrated that Diazoxide, synergistically used with erythropoietin, improves the clinical outcome in spinal cord ischemic injury. Diazoxide and erythropoietin individually displayed modest motor function and viable neurons compared to the vehicle group. However, when combined, substantial preservation of hind limb motor function and cytoarchitectural changes in the anterior horn was observed compared to the control group. Moreover, the effect of both drugs leads to a prominent pathway activation of CREB and STAT3 compared with other groups [110]. Moreover, when Diazoxide and erythropoietin treatment were administered together, nerve growth factor (NGF) was upregulated. NGF attenuated spinal cord ischemic injury by preserving motor function and increasing neuronal viability compared to other groups [111]. In addition, Diazoxide was reported to amplify the antiapoptotic effect of erythropoietin by enhancing βcR expression [112].

**Glibenclamide** (Glyburide, DiaBeta), also known as glyburide, is a common medication used to treat diabetes mellitus type 2. In 2007, Simard and colleagues reported that sulfonylurea receptor 1 (SUR1)-regulated Ca^2+^-activated [ATP]i-sensitive nonspecific cation (NCCa-ATP) channels of the capillary endothelium in the spinal cord are key to capillary fragmentation following SCI [113]. Through the blockage of NCCa-ATP channels with the FDA-approved anti-diabetic glibenclamide, Simard et al. observed improved behavioral outcomes, decreased lesion volumes, and significant white matter preservation in a rat model of unilateral cervical SCI [113]. Recently, an initial open-label pilot phase study entitled “Spinal Cord Injury Neuroprotection with Glyburide: a pilot, open-label, multicenter, prospective evaluation of oral glyburide in patients with acute traumatic spinal cord injury in the USA” (trial registration numbers NCT02524379 and 2014H0335) was initiated in order to assess the safety and feasibility of administering oral glyburide in patients in the acute phase after traumatic cervical SCI. Patients had an oral drug regimen, which begun within 8 h of injury and continued for 72 h at a daily dose of 3.125 mg on day 1 and 2.5 mg on day 2 and day 3. The screening trial was estimated to be completed in June 2022 [114]. At the time, a total of 24 patients were screened throughout the study to confirm eligibility. Unfortunately, data were not analyzed, as the study was abandoned since the principal investigator left the university.

The initial mechanical damage combined with the ionic imbalance and the excessive release of glutamate to the extracellular space will promote **excitotoxicity** and further neuronal death. The modulation of this secondary event could result in the preservation of neurons and oligodendrocytes and, consequently, lead to motor recovery.

**Levetiracetam** (LEV) is an antiepileptic drug that binds to synaptic vesicle protein SV2A, interfering with presynaptic neurotransmitter release. The ability to selectively increase the expression of glutamate transporters, thus subsiding glutamate excitation, suggests LEV as a potential modulator of neurotoxicity present in SCI [115]. A thoracic and cervical SCI model was used in Wistar rats to assess LEV’s therapeutic potential. Animals treated with LEV presented remarkable functional recovery. When treated with LEV, SCI rats could support their body weight, had better locomotor velocity and rearing activity, and were able to perform occasional plantar steps. Molecular analysis showed that LEV reduced lipid peroxidation and was able to modulate glutamate excitotoxicity. Histological analysis revealed that LEV treatment promoted a marked increase in motor neurons survival, axonal preservation, and a reduction in the cavity size in the lesion site. Further evaluation of its neuroprotective effect showed a reduction in excitotoxicity pathways, which promoted the survival of motor neurons, oligodendrocytes, and motor fibers tracts, as well as a decrease in microglia/macrophages activation in the lesion site in both models [115]. Moreover, a recent study in a SCI mouse model demonstrated that Levetiracetam attenuates spinal cord injury by suppressing the expression of perforin. Their results showed that animals treated with LEV had a restored BSCB, a reduction in the apoptosis rate of the nerve cells in the trauma area, and an improved hind limb motor function, characterized by the protection of nerve conduction and walking coordination [116]. The effect of Levetiracetam in the inflammatory response after SCI is not yet totally clear, with one study not demonstrating any effect [115] and another showing the inhibition of several inflammatory factors by LEV [116].

The 2-amino-6-(trifluoromethoxy)benzothiazole (**Riluzole**, Rilutek^®^) is a member of the benzothiazole class indicated for the treatment of patients with amyotrophic lateral sclerosis (ALS) [117]. Riluzole is a Na^+^ channel blocker, and its neuroprotective effects over the spinal cord are exerted on neurons by contradicting the increase in Na^+^ concentration and reversing the operation of axonal Na^+^/Ca^2+^ exchangers. Moreover, by reducing the excessive influx of sodium, riluzole can also reduce glutamate release and attenuate excitotoxicity [118]. Aside from in vitro studies, riluzole can enhance axonal conduction, prevent cellular necrosis and apoptosis, and potentiate nerve fiber regeneration [118]. Intraperitoneal administration of riluzole (4 mg/kg) in rats after SCI promoted recovery of locomotor function, decreased levels of inflammatory cytokines and M2-like polarization of microglia/macrophages [119], reduction in central cavity size of the spinal cord, and improved neurological functions [120]. In compliance, several studies have demonstrated that riluzole has a neuroprotective effect, improving neurologic function recovery, containing the spread of injury in SCI, and decreasing neuropathic pain [121,122,123].

Fehlings’ lab is actively working with this compound, having already published several works with pre-clinical data and begun clinical studies. A phase I clinical trial was conducted to evaluate the safety and pharmacokinetics of riluzole in 36 acute SCI patients [124]. Participants were randomized in a 1:1 ratio to riluzole or placebo. The trial focused on a more homogeneous population of cervical SCI. Patients received the treatment within 12 h after injury. Patients in the experimental arm received 100 mg of riluzole every 12 h for two doses in the first 24 h post-injury, and 50 mg twice daily for the following 13 days. Medication was administered orally or by nasogastric tube. This trial showed some improved ASIA motor scores associated with the treatment and provided the impetus for a phase IIb/III double-blinded, placebo-controlled trial [Riluzole in Acute Spinal Cord Injury Study (RISCIS)] [125,126]. In the phase III trial, 351 patients were enrolled and randomized in a 1:1 ratio to riluzole or placebo. Inclusion criteria and temporal drug administration were maintained in the distinct phase trials. The current phase III study had the purpose of evaluating riluzole in improving motor outcomes as well as evaluating its efficacy on sensory recovery, functional outcomes, quality of life outcomes, and health utilities [127]. Results from the study are still not yet available.

A growing body of evidence demonstrates that **hormone-related therapies** support neuroprotection after SCI. Although progesterone and estrogen are gonadal steroid hormones, their actions are not restricted to reproductive functions, since there is evidence of their effect on the central and peripheral nervous systems during development and in adult life either in health or after injury.

**Progesterone** is an endogenous steroid that has a variety of important functions in the body. It is also a crucial metabolic intermediate in the production of other endogenous steroids, and plays an important role in brain function as a neurosteroid [128]. The presence of sources and receptors of progesterone within the CNS, as well as its modulation of inhibitory and excitatory amino acids, indicate a possible broader role for progesterone than simply as a gestational hormone [129,130,131]. The administration of progesterone demonstrated it to be neuroprotective in intracerebral hemorrhage (ICH) by inhibiting neuroinflammation, promoting axonal regeneration, and reducing cell apoptosis, myelin loss, neutrophil infiltration, microglia, and astrocyte activation in an animal model [132]. Several studies have shown that progesterone regulates some key features of neuronal function after SCI [133]. In spinal cord injury, progesterone modulates the galaninergic and NPYergic systems associated with neuropathic pain [134], upregulates the oligodendrocyte differentiation program, and increases the number of TGFβ1 positive astrocytes and microglia cells [135]. Yang et al. have also shown that progesterone attenuates axonal dieback, reduces neuronal death, accumulates astrocytes and microglia, and downregulates pro-inflammatory cytokines [136]. However, some results are contradictory regarding the administration of progesterone in SCI. Cavalcante and colleagues demonstrated that progesterone could not prevent nor attenuate spinal cord ischemic injury, revealing no differences in motor function, viable neurons, or cell apoptosis [137].

**Estrogen** is a steroid hormone explored as a potential neuroprotective drug for the injured spinal cord. Estrogen presents a wide range of cellular and extracellular effects, mediated by estrogen receptor (ER) α and β related mechanisms [138]. Regarding neuroprotection, estrogen promotes neurite outgrowth, neuron sprouting, and synaptogenesis, and also increases levels of acetylcholine, density of NMDA receptors, and NGF expression [138,139,140]. These effects require a long activation time, and fail to explain estrogen’s neuroprotective effects when administered immediately before or after inducing brain injury. In SCI, estrogen was administered in a wide range of doses, from 0.1 mg/kg to 600 mg/kg, demonstrating a dose effect in some studies [141,142,143]. Evidence has documented that estrogen treatment after SCI improved locomotor activity, reduced the expression of several inflammasome components, and activated microglia and oligodendrocytes, resulting in the attenuation of neuroinflammatory processes [144]. Cox et al. demonstrated that nanoparticle-based estrogen delivery increased axonal regeneration, VEGF, and glial-cell-derived neurotrophic factors, and also improved locomotor and bladder functional recovery whilst reducing post-injury lesion sites, reactive gliosis, and glial scar formation [145]. A similar study, by Haque and colleagues, revealed that in vivo low doses of estrogen (40 nM) attenuated ROS production and calpain activity in microglia, astroglia, macrophages, and fibroblasts cells. They have also shown that estrogen decreases vimentin immunoreactivity, implicating a possible regulation of fibroblast activation by this hormone. In vivo, focal delivery increased tissue distribution of estrogen, altered Bax/Bcl-2 ratio, and attenuated demyelination in the injured spinal cord. Interestingly, while fast release of nanoparticles of estrogen reduced the Bax/Bcl-2 ratio, a slow release was effective in reducing glial activation and penumbral demyelination distal to the lesion site in SCI [146].

As previously referenced, a series of metabolic alterations occur on the spinal cord tissue after injury, resulting in more cell death and neurodegeneration. In order to counteract these **metabolic alterations**, molecules such as statins or polyunsaturated fatty acids have been widely tested in the SCI context. Below, we will briefly describe the main findings on the usage of molecules aimed to modulate these metabolic alterations.

**Atorvastatin** is a member of the statins firstly synthesized in 1985 by Bruce Roth [147]. Sometimes known as Lipitor, atorvastatin lowers plasma low-density lipoprotein (LDL) cholesterol levels by inhibition of 3-hydroxy-3-methylglutarylcoenzyme A (HMG-CoA) reductase. In acute SCI context, atorvastatin was evaluated in a 5 mg/kg dosage administered via oral gavage [148] and by intraperitoneal injection [149]. Atorvastatin revealed neuroprotective effects by decreasing biochemical markers of oxidative stress, mild glial cell infiltration, p53, and MMP-9, increasing spare axons [150], and upregulating MBP levels [151]. In addition, Haazza and colleagues also found atorvastatin to improve hindlimb function, sensory response to noxious stimuli, and placing/stepping reflex in a model of ischemia-reperfusion injury of the spinal cord. Regarding non-behavior outcomes, it was shown that atorvastatin reduced proinflammatory cytokines, oxidative stress, strong expression of Bcl-2, and mild expression of Bax in neuronal cells. Furthermore, histopathological alterations under treatment presented loss of polarity and decrease in basophilia of cytoplasm, yet also mild vacuolization of white matter. GFAP-immunostained spinal cord segments exhibit neuronal cells with moderate glial cell infiltration and vacuolization. Haazza and colleagues also noted that in general, combined treatment of both atorvastatin and L-carnitine ameliorated the neurological, biochemical, and histological alterations [152]. Bimbova et al. revealed that a single dose of atorvastatin (5 mg/kg, i.p.) could decrease the levels of IL-1β and the infiltration of macrophages in both white and gray matter, as well as inhibition of M1 and M2 phenotypes. Moreover, atorvastatin significantly decreases the expression of caspase-3 in the oligodendrocytes, astrocytes, and neurons around the lesion site. In this study, atorvastatin treatment promoted higher expression of neurofilaments in the dorsolateral part of the spinal cord, axons outgrowth along the craniocaudal extension, and GAP43-positive fibers in the lateral column around the epicenter. Regarding motor function, a significant improvement was observed from day 30 to 42 [153]. Although studies in animal models have revealed atorvastatin as a potential therapy for spinal cord injury, a cohort study with 60 patients revealed no improvement at the 3- and 6-month follow-up in patients that were administrated atorvastatin. Nonetheless, when comparing the two in pain severity, the group treated with atorvastatin had a better outcome [154].

**Resveratrol** is a polyphenolic compound synthesized by plants in response to stress, injury, and infection [155]. This secondary metabolite gained popularity for acting as an anti-aging and life-extended agent in several animal models [156,157,158]. Moreover, this bioactive compound has many properties, including activity against glycation and anti-oxidative and anti-inflammatory effects, as well as involvement in neuroprotection, cutaneous wound healing, and scarring [156,159,160,161,162]. Several studies have recognized the potential of resveratrol in modulating innate and adaptative immunity by interacting with a wide range of molecular targets. At the molecular level, it targets SIRT-1, AMP, NF-Κb, MAPK, AP-1, AA pathway, inflammatory cytokines, anti-oxidant enzymes, gluconeogenesis, lipid metabolism, mitochondrial biogenesis, angiogenesis, and apoptosis [16,159,161]. Recent evidence has indicated resveratrol as a potential therapeutic drug for SCI due to its ability in controlling inflammation, apoptosis, reducing astrocyte activation, glial scar formation, exerting neuronal protection, and favoring motor function and spinal cord injury recovery [163,164,165,166,167]. In order to explore the mechanisms behind the resveratrol action in SCI, animals received resveratrol (100 mg/kg) immediately after injury. They observed that resveratrol through the SIRT1/AMPK signaling pathway improved motor function recovery, increased survival of motor neurons, and reduced lesion size, as well as inhibiting apoptosis and autophagy [168]. Meng and colleagues also revealed that resveratrol treatment potentiated the activation of autophagy via the AMPK/mTOR pathway, promoting recovery of neurologic dysfunction and suppression of neuroinflammation [169].

**Omega-3** polyunsaturated fatty acids (PUFAs) include atearidonic acid (SDA), docosapentaenoic acid (SDA), and omega-3 α linolenic acid (ALA), as well as its products, eicosapentaenoic acid (EPA) and docosahexaenoic acid (DHA), and the omega-6 ALA and its derivate arachidonic acid [16,170]. Early reports revealed the importance of these polyunsaturated fatty acids in health by their capacity to modulate cell signaling cascades, gene expression, membrane lipid composition, and eicosanoid biosynthesis [170,171]. In fact, PUFAs have been gaining increased attention regarding their biological functions in controlling immunity, traumatic brain injury, and disease [172,173,174,175]. After SCI, several physiological parameters undergo drastic changes, aiding immune dysfunction and chronic inflammation. Taking advantage of PUFAs properties, dietary manipulation may be a possible therapeutic approach to promote a reduction in systemic inflammation in SCI [16,176,177]. Baazm et al. reported that administration of PUFA n3 (250 nmol/kg), immediately after injury and every 24 h for 3 days, improved locomotor recovery and suppression of microgliosis, increased the number of oligodendrocytes, and prevented demyelination by targeting the NLRP3 inflammasome [178]. Administration of omega-3 fatty acid (50 mg/ kg) for 30 days in animals with SCI showed that omega-3 fatty acid supplementation reduced oxidative stress, apoptosis, and the levels of inflammatory markers [179]. Nie and colleagues also revealed that exogenous dietary high n-3 PUFAs inhibited the mTOR pathway by increasing autophagy and functional recovery in rats with SCI [180].

The modulation of the **inflammatory response** after SCI has been one of the most explored events to achieve therapeutic efficacy. Among the candidates to control inflammation are the antibiotic minocycline, non-steroidal anti-inflammatory drugs, and several cytokines. In the last years, more and more evidence has been demonstrating that rather than stopping the inflammatory process observed after injury, the aim should be on shifting the immune response from the persistent pro-inflammatory to an immune response that fosters tissue healing and regeneration [38].

**Minocycline** is a second-generation tetracycline with anti-inflammatory and neuroprotective properties. The biological effects of minocycline include inhibition of microglial activation, caused by reduction in mRNA of both interleukin 1β (IL-1β), and TNF-α. In addition, minocycline exerts an inhibitory effect on the activity of NF-κB in microglia, causing a reduction in inflammatory mediators such as COX-2 and iNOS. Moreover, the inhibitory effect on the activity of iNOS, COX-2, and MMPs exerts neuroprotective functions in CNS injuries [181,182,183,184]. Concerning behavioral outcomes, minocycline presented improved functional scores on the BBB scale, an inclined plane test score, and attenuation of neuropathic pain [185,186,187]. Non-behavioral outcomes observed include a decrease in lesion area, an increase number of descending sympatho-excitatory axons, reduction in local free radicals, lipid peroxidation, and glial fibrillary acidic protein expression, as well an increase in brain-derived neurotrophic factor [185,188]. Pourkhodadad and colleagues revealed that the combination of minocycline with olfactory ensheathing cells graft is more effective to counteract SCI damage by decreasing functional damage, astrogliosis, and cavitation in spinal tissue. The coadjuvant therapy also caused a reduction in IL-1β, TNF-α, caspase-3, MDA, and NO when compared with minocycline or olfactory ensheathing cells alone [187].

In 2012, a phase II, double-blinded, placebo-controlled clinical trial of minocycline for acute traumatic SCI was initiated. Fifty-two patients enrolled in this study. One year later, the treated group had a 6-point greater motor recovery when compared to the placebo group; however, results did not reach statistical significance [189]. This study concluded that minocycline given intravenously within 12 h and 7 days resulted in steady-state serum concentrations reaching the target values present in animal studies. No significant adverse events were identified. The treatment was associated with apparent improvement in neurological and functional outcomes compared to the placebo [190]. Cerebrospinal fluid (CSF) collected from 29 subjects enrolled in the previous phase II trial showed IL-1β, MMP-9, HO-1, and CXCL10 as targets for minocycline and markers of injury severity in SCI [191].

**Immune mediators** are a broad range of small signaling molecules. These molecules, specifically the anti-inflammatory cytokines, including IL-10, intraspinal administration of IL-37, systemic, and cell-mediated delivery of IL-13, were shown to present beneficial effects after SCI [192,193,194].

**IL-10** is a potent anti-inflammatory cytokine synthetized by numerous cell types, such as T-helper cells, monocytes/macrophages, astrocytes, and microglia [195,196]. This cytokine suppresses most monocytes/macrophage inflammatory responses in the peripheral immune system. IL-10 also inhibits the production of multiple cytokines, chemokines, CAMs, and ROS [197,198,199]. Regarding CNS, IL-10 reduces TNF-α production by astrocytes and antigen presentation by both astrocytes and microglia [200], and it prevents experimental allergic encephalomyelitis in Lewis rats [198,201,202]. Bethea and colleagues treated spinal cord injured rats with IL-10 30 min post-injury [201]. IL-10 treatment was able to significantly improve motor function and reduce lesion volume [201]. The authors relate these results to the reduction of TNF-α synthesis in spinal cord tissue and macrophage modulation. These results show that cytokine treatment can be a potent tool for SCI inflammatory modulation.

**IL-4** is a pleiotropic anti-inflammatory cytokine, first recognized as a T-cell-derived soluble factor that stimulates the proliferation of B cells. Aside from T cells, IL-4 is produced by NKT cells, mast cells, basophils, and eosinophils [203,204]. This cytokine plays a crucial role in the regulation of several signaling pathways between immune cells, as well as in the regulation of recruitment, activation, and inhibition of both immune and nonimmune cells [203,204]. In light of the role of IL-4 in the pathogenesis of spinal cord injury, Quijorna et al. demonstrated that after SCI, levels of IL-4 were unnoticeable. They also observed that only one administration of IL-4 48 h after SCI was enough to drive microglia and macrophages from an M1 to an M2 phenotype, enhancing functional outcomes and reducing tissue damage [205]. On the other hand, Lima and colleagues analyzed the effect of systemic delivery of IL-4 for 7 days in a rat model of contusion SCI. They noticed that IL-4 leads to increased levels of IL-10 and reduced inflammatory markers. In addition, administration of IL-4 altered microglia/macrophage morphology, potentiated the number of motor neurons and oligodendrocytes, and allowed recovery of weight support. Although no significant motor recovery was seen, IL-4 emerged as a neuroprotective agent with potential to be co-integrated with other therapeutic approaches in SCI [206].

**Maresin** is a pro-resolving lipid mediator first identified in human macrophages, which was active in inflammatory processes [207]. Studies have demonstrated that Maresin exerts strong anti-inflammatory action capable of modulating various actions on immune cells contributing to the resolution of acute inflammation and organ protection [207,208,209]. Recent evidence indicated that exogenous administration of Maresin could resolve inflammatory response after SCI by silencing crucial inflammatory pathways, reducing pro-inflammatory cytokines, shifting macrophages phenotype towards a reparative profile, potentiating clearance of neutrophils by macrophages, and reducing macrophages in the lesion site. Regarding functional recovery, Maresin administration improved locomotor recovery and limited secondary injury progression [210]. Maresin is a promising therapeutic approach for recovery in spinal cord injuries due to its strong anti-inflammatory response and functional neurologic response.

Although, technically, **hypothermia** is not a molecular therapy, we decide to include it here due to its effects on neuroprotection after SCI. The intentional induction of hypothermia can be achieved through systemic and endovascular (local) approaches. The systemic hypothermia can be achieved either by surface cooling or internally through endovascular cooling [211]. Surface cooling with ice packs applied to the patient’s body or gastric lavage with ice-cold fluid is the simplest method to induce hypothermia [212].

More rapid cooling can be achieved with an endovascular approach with the infusion of cold saline or with intravascular or extracorporeal cooling devices. This endovascular temperature regulation technology has recently received FDA approval [213]. Briefly, this device employs a central venous catheter inserted via the femoral or subclavian veins following the standard central venous catheter placement technique. The devices contain a heat exchanger, through which cooled (or warmed) saline circulates. This will result in the temperature transfer between the catheter and the circulating blood. At no point does the saline leave the system and enter the circulatory system [211].

Systemic hypothermia has been shown to reduce inflammatory cell infiltration, myeloperoxidase activity, and vasogenic edema, increase the stabilization of BSCB, and limit cell death [214]. Despite these benefits, systemic hypothermia may have some side effects, including bradycardia, respiratory infections, and deep vein thrombosis. On the other hand, local hypothermia of the spinal cord avoids many of these concomitant issues. In 2014, Hansebout and colleagues reported improved recovery among cervical and thoracic patients after acute local hypothermia (within 8 h of injury) [215]. Similarly, a pilot study of systemic hypothermia in patients with cervical complete SCI demonstrated fewer adverse effects and a trend toward improved recovery when induced within 9 h of trauma [216]. Considering these results, a follow-up phase II/III randomized clinical trial (ARCTIC, NCT02991690) was initiated in May 2017 to evaluate complications specifically associated with this therapeutic intervention [217]. In this prospective multicenter case-controlled study of systemic hypothermia in acute cervical SCI, patients received modest endovascular hypothermia for 48 h. Respiratory complications, namely respiratory failure followed by pneumonia, were the commonest drawback associated with acute SCI. Cardiac adverse events, particularly bradycardia, are also common complications observed in patients. These preliminary data show no difference regarding complication incidents of a particular group within the first 6 weeks after acute cervical SCI [217].

In addition to the previously referenced events, SCI research has also focused on the modulation of the spinal cord milieu to create a more permissive environment for **axon regeneration**. Different strategies have been explored in order to achieve functional recovery; among them, the blockage of inhibitory molecules (Anti-Nogo-A) and enzymatic degradation (Chondroitinase ABC) of scar tissue have been the most successful so far.

After injury, CNS neurons have a limited regenerative capacity. As previously described, this is in part due to myelin-associated neurite growth inhibitors such as Nogo-A [218,219]. **Anti-Nogo-A** is a monoclonal antibody against this inhibitory molecule. This antibody has been widely tested as a therapeutic approach to promote axonal regeneration after SCI. It has demonstrated efficacy both in rodent models [220,221,222] and in primate models of cervical SCI [223,224]. The anti-Nogo-A antibody has been shown to promote axonal sprouting and to improve functional recovery following injury. After sectioning the corticospinal tract in adult rats, neutralizing Nogo-A with monoclonal antibodies leads to axonal regrowth and compensatory sprouting, along with increased motor recovery [220,225,226]. Chen and colleagues describe that combined therapy regarding sequential administration of Anti-Nogo-A antibody and intense locomotor training improved step consistency and reduced toe dragging and climbing errors. They also noted that treated animals adopted a more parallel paw position in bipedal walking and a better quadrupedal locomotor [227]. The development of neurogenic lower urinary tract disorder is a common feature in spinal cord injury patients, and Anti-Nogo-A antibody treatment has shown positive benefits regarding this condition. Intrathecal administration of Anti-Nogo-A antibodies over 2 weeks in incompletely spinal cord injured rats led to a significative reduction in urodynamic abnormalities. However, no treatment effect was observed when this therapeutic approach was evaluated in animals with complete SCI [228]. Considering the extensive pre-clinical data on its therapeutic effect, a non-randomized, open-label, phase I clinical trial of humanized anti-Nogo-A antibody (ATI-355; Novartis Pharmaceuticals) was initiated in 2006. Anti-Nogo-A was delivered intrathecally in sub-acute SCI (4–14 days after injury) in order to assess its feasibility, tolerability, and safety [229]. In total, 52 SCI patients with trauma between C5 and T12 levels were recruited for the study. The results demonstrated that anti-Nogo-A was well tolerated in humans, and that using intrathecal antibody administration may be considered in future SCI trials [229]. A phase II clinical trial, entitled “Nogo Inhibition in Spinal Cord Injury” (NISCI–NCT03935321), began in 2019 to test the efficacy of the anti-Nogo-A antibody treatment after SCI in severely injured paraplegic and tetraplegic patients [230]. This study is estimated to be completed in 2023.

**Chondroitinase ABC** (ChABC) is an enzyme obtained from the bacteria lysate of Proteus vulgaris [231]. The use of ChABC in SCI has been widely explored [232,233,234]. ChABC’s therapeutic effect after SCI is due to its ability to degrade the sugar chains from CSPGs [235]. This allows the enzyme to degrade inhibitory molecules to axonal regeneration and to break down the PNNs. As previously referred, PNNs inhibit nerve regeneration, and their removal can result in new nerve connections. Through the breakdown of CPSGs chains [232], ChABC was able to promote functional recovery of SCI rats [232]. It is also capable of promoting neuronal plasticity, allowing undamaged neurons to sprout and assume functions of damaged neurons. This feature is particularly relevant in cases of incomplete SCI. The regenerative capacity of descending axons through the lesion after complete spinal cord transection was investigated by Takiguchi and colleagues. The authors noted that administration of ChABC at the lesion site after complete spinal cord transection promoted the passage of descending 5-TH axons through the lesion site and recovery of locomotor function [236]. Aside from its potential neuroregenerative action, ChABC has been studied regarding its prospects in inhibiting neuropathic pain. Janzadeh et al. documented that intraspinal injection of ChABC or combined with photobiomodulation therapy leads to decreased pain-related factors, increased anti-nociceptive factors, and improved movement [237]. Studies also demonstrate that ChABC, when combined with peripheral nerve grafts, restores urinary and respiratory functions [238,239]. Regarding its therapeutic window of efficacy, García-Alías and colleagues performed a study wherein treatment began immediately after injury or at 2, 4, or 7 dpi [240]. Results support the possibility that ChABC treatment can be successfully applied starting up to seven days after SCI [241]. Pre-clinical studies have demonstrated the therapeutic potential of ChABC treatment following SCI. However, the main issue with ChABC therapy is that it may require multiple injections [242]. Recently, researchers at the University of Cambridge created a version of ChABC that can be expressed by human cells and delivered by gene therapy [243,244]. This could improve efficacy and facilitate clinical translation.

Recently, ChABC combined therapies have been explored to enhance SCI regeneration. Raspa et al. demonstrated that self-assembling peptides enhanced stability and prolonged in vivo delivery of ChABC, favoring host neural regeneration and behavioral recovery in chronic SCI [245]. Prager and colleagues studied the possible impact of combined canine mucosal olfactory ensheathing cells and ChABC in an SCI rat model. The combined treatment leads to a modest functional improvement [246]. Prager et al. conducted a prospective, single-arm clinical safety study in dogs with chronic SCI in order to characterize the feasibility and safety of the treatment, regarding the use of olfactory ensheathing cells as a combined therapy. Although this combinatory approach was considered feasible and safe with a reduced number of animals, no functional improvement after transplantation was observed [247]. Another strategy that has emerged as promising to regenerate SCI is the combined delivery of ChABC with cell-based therapies. Nori et al. investigated the therapeutic potential of XMC hydrogel containing ChABC combined with neural progenitor cells (NPC) which were reprogrammed towards an oligodendrogenic fate (oNPCs). The combined treatment enhanced the survival of oNPCs surrounding the lesion epicenter, oligodendrocyte differentiation, and remyelination of the spared axons. This synergic approach promoted synaptic connectivity of anterior horn neurons toward an oligodendrogenic fate (oNPCs), improved motor function, and did not exacerbate neuropathic pain after chronic SCI [248]. Suzuki and colleagues also demonstrated that previous treatment with ChABC could positively modulate the spinal cord microenvironment, enhancing the integration of transplanted NSCs and forelimb recovery. Finally, Führmann and associates evaluated the combined delivery of chABC with human iPSCs-derived neuroepithelial stem cells (NESCs). ChABC had the capacity to influence the injured microenvironment, supporting neuronal survival and differentiation. Cell transplantation had an impact regarding cavity formation; however, neither individual nor combined treatment influenced the behavioral outcome [249].

**Table 1:** Molecular therapies for spinal cord injury.

Therapeutic Agent	Molecular Target	Mechanisms of Action	Reference
Diazoxide	K-ATP Channels	Aids spinal cord ischemia-reperfusion injury by decreasing apoptosis and preserving neuronal viability and motor function. The synergistic effect with erythropoietin contributes to higher therapeutic effects.	[109,110,111,112]
Glibenclamide	NCCa-ATP	Acts as an NCCa-ATP antagonist, improving behavioral outcomes, decreasing lesion volume, and significantly preserving white matter.	[113]
Levetiracetam	SV2A	Reduces glutamate excitotoxicity, lipid peroxidation, and apoptosis, and improves astrocitic function. Significant functional and histological improvements were observed.	[115,116]
Riluzole	Na^+^ Channels	Reduces glutamate excitotoxicity and inflammatory cytokines, and induces a less active state in microglia and macrophages. Decreases cavity size and neuropathic pain, and improves motor function recovery.	[118,119,121,122,123]
Progesterone	PR	Increases TGFβ1 positive astrocytes and microglia cells. Attenuates axonal dieback, neuronal death, and pro-inflammatory cytokines. Contradictory results were observed.	[134,135,136,137,250]
Estrogen	ER-α and ER-β receptors	Attenuates the expression of several inflammasome components, apoptosis, and ROS production. Reduces glial scar formation and demyelination, and improves axonal regeneration.	[138,144,145,146]
Atorvastatin	HMG-CoA reductase inhibitor	Reduces oxidative stress markers, pro-inflammatory cytokines, and apoptosis. Improve motor functions and increase spare axons. Synergic treatment with and L-carnitine enhances therapeutic outcomes. No improvements were observed when tested in humans aside from a decrease in neuropathic pain.	[148,150,151,152,153,154]
Resveratrol	Pleiotropic interactions	Activates autophagy and inhibits apoptosis and pro-inflammatory cytokines. Reduces astrocyte activation and glial scar formation. Improves motor function and survival of motor neurons.	[161,163,164,165,166,167,168,169]
Omega-3 fatty acids	Pleiotropic interactions	Reduces oxidative stress, apoptosis, and inflammatory markers, and increases autophagy. Inhibits microgliosis and demyelination and increases oligodendrocytes. Locomotor recovery was observed.	[16,176,177,178,179,180,251]
Minocycline	Pleiotropic interactions	Improves behavior outcomes. Reduction in free radicals, lipid peroxidation, glial fibrillary, and acidic protein expression, as well as an increase in brain-derived neurotrophic factor were observed. Synergic effects with olfactory ensheathing cells graft were observed.	[185,186,187,188]
Il-10	IL-10R1 and IL-10R 2	Improves motor function, reduces lesion volume, decreases TNF-α levels and modulation of macrophages.	[252,253]
IL-4	IL-4Rα	Reduction in tissue damage and inflammatory markers were observed. Favors macrophage and microglia phenotype to a pro-regenerative phenotype. Improves locomotor recovery.	[205,206,254]
Maresin	RORα/LGR6	Reduces pro-inflammatory cytokines, induces pro-regenerative macrophages phenotype, improves neutrophil clearance, and reduces macrophages in the lesion site. Improves locomotor recovery.	[210,255]
Hypothermia	-	Reduces inflammatory cell infiltration, cell death, MPO activity, and vasogenic edema, and increases BSCB stabilization.	[214]
Anti-Nogo A	Nogo-A receptor	Promotes axonal regrowth and sprouting. Improves functional recovery, and reduces urodynamic abnormalities.	[220,225,226,227,228]
ChABC	CSPGs	Promotes functional recovery, neural plasticity, and regeneration. Synergic treatment with cell therapies.	[232,236,237,245,246,247,248,249]

### 4.2. Cell-Based Strategies

The transplantation of cells to promote SCI repair is widely explored in several labs around the globe. Cells are used either to provide the lesion site with relevant CNS populations that will replace dying neurons, oligodendrocytes, or astrocytes, to help to reestablish lost connections, or as sources that guarantee trophic support and enhance neural regeneration [256]. Cell-based therapies have been demonstrated to promote functional recovery after SCI in a wide range of experimental animal studies [257,258,259].

**Embryonic stem cells** (ESCs), derived from the blastocyst, comprise two crucial properties: pluripotency and self-renewal ability. The pluripotency that confers the ability to differentiate into cells from all three germ layers makes this cell type an attractive source for a variety of applications [260]. Apart from the ethical issues, the main challenge in using ESCs is the correct cues to direct differentiation of the specific desired cell types and simultaneously prevent teratoma formation [257]. ESCs-based approaches for SCI treatment have been mainly based on the differentiation between these cells and Neural Stem Cells (NSCs). Keirstead et al. transplanted NSCs obtained from mouse ESCs into a rat spinal cord after thoracic SCI. Most transplanted cells survived, migrated away from the injury site, and were shown to preferentially differentiate into oligodendrocytes and astrocytes [261]. Kakinohana et al. demonstrated that the injection of human ESC-derived neural precursor cells (hNPCs) into the ischemia-injured lumbar spinal cord of rats and lumbar spinal cord of naïve immunosuppressed minipigs resulted in successful engraftment of hNPCs and their maturation into neurons [262].

The clinical application of embryonic-derived stem cells in SCI patients started with the FDA approval for a Phase I clinical trial, using oligodendrocytes precursor cells (OPCs) and was performed by Geron’s company in 2009. The rationale for using OPCs therapy was that remyelination (by oligodendrocytes derived from human ESCs) of the spinal cord axons may improve nerve conduction and, thereby, locomotor recovery. Surprisingly, in November 2011, the trial was discontinued due to financial concerns [263]. Geron’s program was terminated later in the same year; nevertheless, to date, no safety issues were reported in the five patients subjected to OPCs transplants [264]. In 2016, Shroff published a retrospective study of a cohort of 226 SCI patients who were also treated with OPCs derived from hESCs in India [265]. The OPCs treatment in paraplegic and quadriplegic patients promoted gains in voluntary movement in areas below injury level, improvement in bladder sensation and control, and gait and hand grip [266]. Tractography analysis, obtained by magnetic resonance imaging (MRI), showed axonal regeneration promoted by the treatment. Moreover, none of the patients in the study developed teratomas or an immune rejection of the transplant; the adverse effects observed were mild, and resolved without sequels reported [265].

There is a controversial discussion concerning human ESCs research, aside from technical issues related to ESCs therapy, which is due to the use of human oocytes and human embryos [267]. **Induced pluripotent stem cells** (iPSCs) emerged as a possible alternative to obtain pluripotent cells directly from adult tissues for autologous transplantation. The iPSCs technology resulted from a pioneer work developed by Yamanaka’s lab in Japan in 2006, which showed that the introduction of four transcription factors reverted the phenotype of differentiated adult cells into pluripotent stem cells [268].

A preclinical study investigated the therapeutic potential of transplanting NSCs derived from murine and human iPSCs into a nonhuman primate model of contusive SCI. Similarly to previous rodent studies, the grafted cells were found to survive and differentiate into neurons, astrocytes, and oligodendrocytes, without evidence of tumor formation. In addition, there was an enhancement in axonal sparing/regrowth and angiogenesis at the lesion site, and the prevention of the lesion epicenter demyelination. The treatment also promoted functional recovery of the SCI animals [269]. NSCs derived from iPSCs are currently the most promising source of cells to treat the injured spinal cord. Indeed, based on the amount of pre-clinical data demonstrating efficacy and safety, a clinical trial using these cells has been planned in Japan [270].

Herein we only focused on pluripotent stem cells; however, numerous cell types have been tested for their capacity to repair the spinal cord. Some of them demonstrated promising pre-clinical results and were tested on SCI patients, as is the case of Schwann Cells [271,272], Olfactory Ensheathing Cells [273], Mesenchymal Stem Cells [274] or their secretome [275,276,277], and Macrophages [278,279,280].

### 4.3. Biomaterials

Chronic spinal cord lesion usually leads to the formation of cystic cavities. In this case, the growing axons encounter both chemical inhibitory elements and a physical gap; thus, it is essential to reconstruct the damaged tissue by a growth-promoting environment that would bridge the lesion and stimulate host tissue regeneration. One feasible approach is to fill the cavity with a bioengineered scaffold that can provide structural and/or chemical support for axonal regrowth, and concomitantly serve as a matrix for delivering cells or bioactive molecules.

The design and fabrication of scaffolds for SCI need to take into consideration the mechanical properties of the native spinal cord: favorable surface chemistry, sufficient pore size, porosity, and surface area for cell loading and cell surface interaction, as well as axon regrowth, nutrient transport, and a biodegradation profile that produces adequate residence time [281]. Due to their physical properties, **hydrogels** may be the most adequate biomaterial to repair soft tissues such as nervous tissue [282]. An advantage of some hydrogels is that they can be designed for in situ polymerization and/or cross-linking, therefore needing minimally invasive approaches.

Numerous natural and artificial materials have been tested for their efficacy in repairing the injured spinal cord. **Natural-derived biomaterials** provide structures similar to the natural ECM, which may favor the integration of the implanted scaffold with the host tissue [283]. Natural biomaterials such as collagen [284], hyaluronic acid [285], agarose [286], alginate [287], and gellan gum [288,289] have been showing some promising results for SCI repair.

**Synthetic materials** may also play an important role in SCI tissue engineering, due to their ability to be modified in a controlled fashion to achieve the desired material properties [290]. Among synthetic materials, the multi-channel guidance tubes have drawn much attention among researchers, as these cylindrical multi-channels may guide regenerating axons [291]. Compared to a single lumen tube, the multi-channel scaffolds provide more surface area, allowing for more cell attachment and local release of incorporated growth factors. However, in one study by Wong and colleagues where different macro-structures were tested, the authors observed that both the cylinder tube and the multi-channel scaffolds resulted in a doubling of defect length due to secondary damage, and led to larger scar and cystic cavity formation with no neuronal tissue regrowth. On the contrary, open-path structures were able to facilitate axonal regeneration through the lesion site and limit secondary injury progression [292]. It is important to point out that this is relatively new field; thus, there is no consensus yet in the literature on the optimal characteristics of biomaterials for spinal cord regeneration and repair.

Limitations in both natural and synthetic materials presented shortcomings regarding application after SCI. Combining materials with different properties may be the answer to developing nerve guidance scaffolds with the biochemical and functional characteristics required for tissue engineering applications. For instant, a hybrid sponge that consists of collagen and polyglycolide fibers has been used in human patients with peripheral nerve injuries since 2002 [293]. Further developments in the physical and chemical manipulation of biomaterials may lead to more axonal regeneration, particularly in combination with other therapeutic strategies, such as cell transplantation and bioactive molecules [294].

### 4.4. Novel Neurorehabilitation Protocols

At the beginning of the 20th century, Philippson [295] and Sherrington [296] reported unexpected observations that revolutionized the conceptions of the neural control of movements. They demonstrated that after a complete transection of the thoracic spinal cord in cats and dogs, the hindlimbs could still preserve a range of motor patterns in response to changing sensory inputs [296]. Graham Brown also demonstrated that the spinal cord exhibits neural circuitries, with an intrinsic capacity to generate rhythmic outputs [297]. A novel therapeutic strategy emerged from the ability to potentiate this intrinsic capacity with successive external inputs.

A severe spinal cord lesion significantly compromises the degree of sustainable excitability in lumbosacral circuitries [298]. Consequently, in the past decade, much effort has been put into the development of strategies to tune the physiological state of lumbar spinal circuits to a level sufficient for stepping and standing to occur. The groundbreaking pre-clinical work performed in the Reggie Edgerton Lab was quickly tested in clinical settings. Angeli and colleagues presented evidence for recovery of independent walking over the ground of patients with complete paralysis. Implantation of an epidural stimulator combined with intensive training resulted in the recovery of intentional walking over the ground in 2 of 4 patients. The other 2, although not able to reach independent bilateral stepping and transition over ground walking, were capable of standing and sitting independently [299]. Gill and coworkers also investigated the effect of epidural electrical stimulation (EES) under multimodal rehabilitation (MMR) in a patient with complete sensorimotor paralysis for 43 consecutive weeks. Previously, that patient had demonstrated the ability to control step-like activity once suspended vertically with the help of a body weight support system, or when side lying. During the training period, MMR combined with EES allowed the participant to independently stand, step on a treadmill, and step over the ground using a front-wheeled walker with trainer assistance [300]. Recently, Wagner and colleagues developed a new neurotechnology consisting of an implant pulse generator capable of real-time control over independently adjusting EES trains to the spinal cord. They noticed that rehabilitation improved walking with EES as well as neurological recovery. After rehabilitation, participants regained voluntary control of walking even without stimulation [301].

EES was also demonstrated to have a positive effect on autonomic functions. For instance, Harkema et al. explored the implementation of epidural stimulation targeted for cardiovascular function (CV-scES). All patients demonstrated improved orthostatic tolerance and cardiovascular response during CV-scES training. These results suggest that CV-scES by itself has an inherent capacity related to adaptive plasticity capable of stabilizing cardiovascular and autonomic regulatory systems [302]. Herrity et al. studied spinal cord epidural stimulation targeting the urinary bladder (UB-scES) in a patient with chronic and motor-completed SCI for a period of 4 months. The results supported the notion that UB-scES also has the capacity to control the urological system [303]. In order to restore hemodynamic stability after SCI, Squair and coworkers developed a neuroprosthetic baroreflex capable of controlling hemodynamics during transient, varying, and sustained orthostatic challenges in the acute and chronic phases of SCI in rodents, non-human primates, and humans. Although this device was validated in one patient with tetraplegia with positive results, future clinical trials are essential to evaluate safety and therapeutic efficacy of the neuroprosthetic baroreflex [304].

EES is currently one of the most promising therapeutic interventions to promote functional recovery after an SCI, however, the mechanisms enabling motor control when delivering neuromodulation therapies and improvements with rehabilitation remain unclear and highly debated [305].

## 5. Conclusions

**Figure 2:**
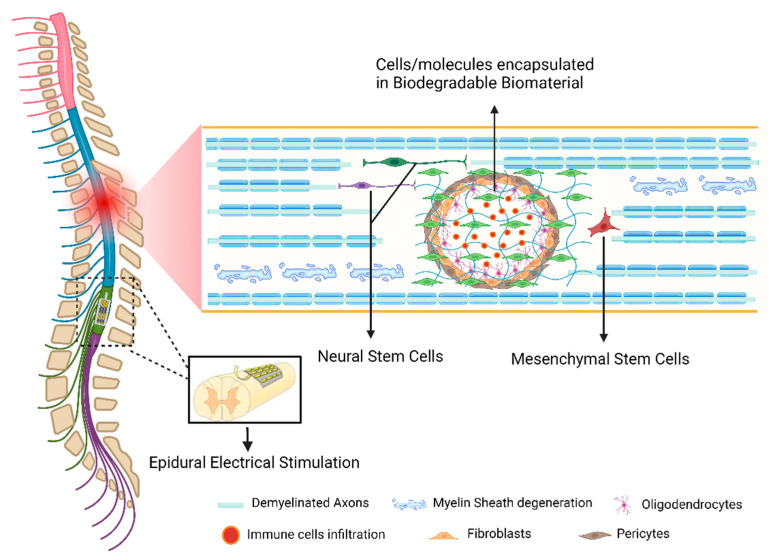
Schematic representation of various regenerative therapies proposed for spinal cord injury repair. Created with BioRender.com (accessed on 11 October 2022).

Despite all of the current advances in SCI therapies, there is not yet an effective treatment available to patients. The acute phase is rich in molecular and cellular events which, if accurately modulated, may lead to protection of neural tissue and functional recovery. For this reason, therapeutic agents administered shortly after the injury have long been explored. However, until now, and despite all the encouraging results observed in preclinical settings, many drugs fail to demonstrate efficacy when tested in the clinical setting. This means that the search for a therapeutic agent that can protect the spinal cord against detrimental events of the “secondary injury” is still open. We may need to rethink how to develop novel and effective therapies for SCI patients. In fact, as aforementioned, the pathophysiology of SCI is a complex and dynamic aggregate of cellular and chemical processes that aim to protect the tissue, but may exacerbate the injury. Thus, a successful strategy might be in the development of new combinatory approaches that can tackle different events (Figure 2), for instance, combining molecular approaches with cell therapy, biomaterials, and/or epidural electrical stimulation.

## Data Availability

Not applicable.
